# Using Fisher information to track stability in multivariate systems

**DOI:** 10.1098/rsos.160582

**Published:** 2016-11-09

**Authors:** Nasir Ahmad, Sybil Derrible, Tarsha Eason, Heriberto Cabezas

**Affiliations:** 1 Complex and Sustainable Urban Networks (CSUN) Laboratory, University of Illinois at Chicago, Chicago, IL, USA; 2 Sustainable Technology Division, Office of Research and Development, US Environmental Protection Agency, Cincinnati, OH, USA; 3 Faculty of Information Technology and Bionics, Pazmany Peter Catholic University, Budapest, Hungary

**Keywords:** Fisher information, data mining, big data, information science

## Abstract

With the current proliferation of data, the proficient use of statistical and mining techniques offer substantial benefits to capture useful information from any dataset. As numerous approaches make use of information theory concepts, here, we discuss how Fisher information (FI) can be applied to sustainability science problems and used in data mining applications by analysing patterns in data. FI was developed as a measure of information content in data, and it has been adapted to assess order in complex system behaviour. The main advantage of the approach is the ability to collapse multiple variables into an index that can be used to assess stability and track overall trends in a system, including its regimes and regime shifts. Here, we provide a brief overview of FI theory, followed by a simple step-by-step numerical example on how to compute FI. Furthermore, we introduce an open source Python library that can be freely downloaded from GitHub and we use it in a simple case study to evaluate the evolution of FI for the global-mean temperature from 1880 to 2015. Results indicate significant declines in FI starting in 1978, suggesting a possible regime shift.

## 1. Introduction

As we pass through the twenty-first century, a massive advancement of information technology and rise of big data affects all the sectors around us, presenting substantial opportunities [1–5]. There is a long history of using information by humans, but in modern days the process has become more advanced and robust [6,7]. Efforts have been made to gain more information not only about observable phenomena but also about latent parameters inherent in system data. Increasing computing power and data availability, coupled with powerful data mining techniques have facilitated the growth and development of a plethora of approaches to discern and capture patterns in system behaviour. Many of these concepts originate from information theory. Rooted in statistics, information theory resides between computer science, mathematics, physics and engineering, and has been widely applied from cryptology to ecosystem dynamics [8,9]. Fisher information (FI), a key method in information theory, offers great promise for data mining applications. It was developed by Ronald Fisher [10] as a means of measuring the amount of information about an unknown parameter that can be obtained by observations. Since then, it has been adapted into a means of monitoring system variables to assess patterns and evaluate stability in system dynamics [11]. FI has been used in a variety of applications from deriving fundamental laws of thermodynamics [12] to assessing dynamic order in real and model systems [11,13–16] to sustainable environmental management and resilience [16–29].

In this paper, we first present a brief overview of FI theory, describe the main calculation algorithm for FI and provide a simple computational example. The FI algorithm was previously coded in Matlab, and the deployed applications are accessible by contacting the code developer directly [18]. Here we present an open source Python code for FI calculations, which is freely available. Furthermore, to demonstrate the use of FI for relatively complex systems, we calculate FI to analyse the evolution of the global-mean temperature from 1880 to 2015.

## 2. Background on Fisher information

FI was first developed by statistician R.A. Fisher [10], as a measure of indeterminacy. In other words, it can be used to measure the amount of information about an unknown parameter, *θ* that is present in observable data. Mathematically, FI, *I*(*θ*), is defined as [11]:

2.1 $$I(\theta )=\int {\frac{\text{d}X}{p_{0}(X|\theta )}\left[\frac{\partial p_{0}(X|\theta )}{\partial \theta }\right]}^{2},$$

where, *p*_0_(*X|θ*) is the probability density of obtaining a particular value of *X* in the presence of *θ*.

In practice, it is essentially impossible to use equation (2.1) because the computation of the derivative of the (∂*p*_0_(*X*|*θ*)/∂*θ*) component is required which depends on the numeric value of the unknown parameter *θ*. Through numerous derivation steps, Mayer *et al*. [16] adapted this equation for application to real systems based on the probability of observing various states of the system *p*(*s*), such that

2.2 $$I=\int {\frac{\text{d}s}{p(s)}\left[\frac{\text{d}p(s)}{\text{d}s}\right]}^{2}.$$

The above equation is the foundational form of FI used in this work. The equation is further simplified by eliminating the complication of handling a small *p*(*s*) in the denominator. To overcome this problem *p*(*s*) is replaced by its amplitude, which is defined as *q*^2^(*s*) = *p*(*s*), thus giving us after some manipulation [16]:

2.3 $$I=4\int {\left[\frac{\text{d}q(s)}{\text{d}s}\right]}^{2}\;\text{d}s.$$

Karunanithi *et al*. [11] further simplified this equation by assuming discrete steps so that d*q* ≈ Δ*q *=*q_i_* − *q_i_*_+1_ and d*s* ≈ Δ*s* = *s_i_* – *s_i_*_+1_. For sequential steps, *s_i_* − *s_i_*_+1_ = 1, equation (2.3) is written as:

2.4 $$\text{FI}\approx 4\sum_{i=1}^{m}{\left[q_{i}-q_{i+1}\right]}^{2},$$

where, *m* is the number of states. A state is defined as a condition of the system determined by specifying a value for each of the variables that characterize its behaviour [11]. Equation (2.4) is used to compute FI numerically for systems characterized by multiple discrete data. The following section will discuss the step-by-step procedure to compute FI. Complete details on the method and related derivations may be found in [11,13,17,20].

## 3. Calculation methodology

Evaluating changes in the probability of detecting different states of a system over time is the foundation of computing FI. Hence, information about a system's condition or state over time is required. A system can first be defined by *n* measurable variables (*y_i_*), which are able to characterize the system and its state at any point in time [11]. The selection of variables is crucial, and effort should be put into selecting variables that are not only pertinent to a system but that also capture critical properties of a system. Each data point *v_i_* at time *t_j_*, (*v_i_*_,*j*_), representing the entire system in a phase space is defined by the set of variables *v_i,j_* = {*y*_1_(*t_j_*), *y*_2_(*t_j_*), … , *y_n_*(*t_j_*)}, to aid in categorizing the system into discrete states. In practice, to measure stability, we note that small fluctuations in a variable do not systematically translate into a regime change. Moreover, some inherent or small measurement error also frequently occurs. We define these fluctuations and small errors as measurement uncertainty which represents random variation in our system data.

Numerically, a parameter Δ*y_i_* is defined as measurement uncertainty such that, if

3.1 $$\;|y_{i}(t_{j})-y_{i}(t_{k})|\leq \Delta y_{i}$$

is true for all variables *y_i_* at time *t_j_* and *t_k_* then the two points are indistinguishable, and they are consequently ‘binned’ together in the same state. In other words, if a system is defined by *n* measurable variables then a state is exemplified as a *n* dimensional hyper-rectangular box, where each side represents the uncertainty for each variable. Here, this set of Δ*y_i_* defines the size of state for the system.

Usually, unless reported with the data, the measurement of uncertainty is unknown. Hence, Karunanithi *et al*. [11] recommend choosing a relatively stable time period in each time series, and then computing the standard deviation (s.d.) of each variable *Y* with population mean *ϑ* and using Chebyshev's inequality, defined by

3.2 $$P(|Y-\vartheta |<k\times \text{s.d.})\geq \left(1-\frac{1}{k^{2}}\right).$$

Equation (3.2) indicates that for any form of a probability distribution, ‘the proportion of the observations falling within *k* standard deviations of the [population] mean (*ϑ*) is at least 1 − 1/*k*^2′^ [30]. Thus, Δ*y_i_* is chosen as ±*k* × s.d. To ensure at least 75% of the data would occur within the level of uncertainty, a *k* of 2 can be selected as 1 − 1/2^2^ = 0.75 [24].

In other words, for one variable, two points can be considered to belong to the same state and be indistinguishable, if they vary within this defined level of uncertainty for this variable. Overall, this means that the state of a system is represented by all the points that are ‘binned’ within a range of uncertainty [11].

As mentioned earlier, the goal of FI is to capture dynamic behaviour in terms of the probability of observing various states of a system. To move through the data, the time period is divided into time windows composed of several time steps (e.g. eight consecutive years), and one measure of FI is calculated for the time window which we attribute to the last time step of the window so that only past data are used in the computation [27]. The time window is then moved by a defined number of steps. Two parameters are, therefore, used to define the moving window, which are the size of the window and the increment of the window. Both of these parameters are expressed in terms of time steps and depending on the data, time steps could be any units of time like years, months, days, weeks, etc. These two parameters are used to move through the data such that the size of the window is greater than the amount of movement for each window in order to capture behaviour that may extend beyond the boundary of the window [24]. The numerical example below illustrates this point. Then, the probability densities *p*(*s*) and eventually, FI for each window are computed. The size of the window depends on the amount of data available, but from empirical tests, a window size of at least eight time steps has been recommended [17]. Further details on the computation algorithm can be found in the US EPA report published in 2010 [18].

After determining the parameters for the integration window (window size, window increment and size of state), the binning process can be initiated [10,24]. To begin, the first point of the time window is selected as the centre of the first state and a hyper-rectangle, whose sides are defined by Δ*y_i_* for each variable of that system, is placed around that point. The points that lie within the hyper-rectangle are binned together. Then, the next unbinned point in the window is taken as the centre of the next hyper-rectangle and similar points found within that hyper-rectangle are binned together. This process continues until all the points in the time window are binned or placed in different states.

**Figure 1.**
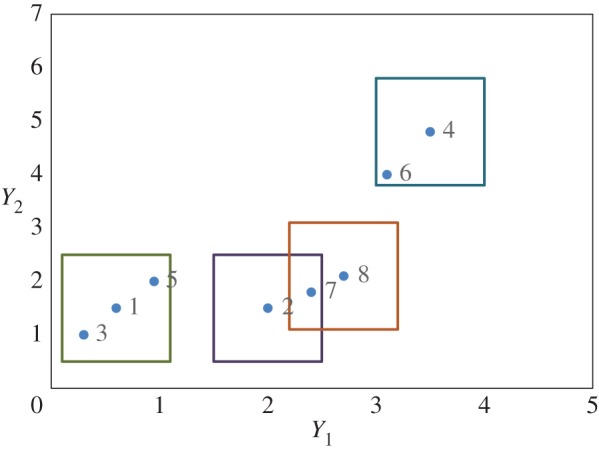
Illustration of the binning process to calculate FI. Note that binning of the points in table 1 resulted in four states.

Following the approach presented by US EPA [18], figure 1 shows the binning process for a system, which is defined by two variables with size of state of 0.5 and 1, respectively (data shown in table 1). In figure 1, we can observe that, first, point 1 is chosen as the centre of the hyper-rectangle and points 1, 3 and 5 binned together to form state 1. Then, point 2, which is the next unbinned point taken as the centre of a new hyper-rectangle and point 2 and 7 binned together to form state 2. After that, the next unbinned point 4 is taken as the centre of a new hyper-rectangle and point 4 and 6 binned together to form state 3. Finally, the only remaining unbinned point 8 forms state 4. Although points 7 and 8 are within the size of state to be binned together, they are not, as point 7 is binned with point 2 earlier. Eight time steps are defined in each window and result in one measure of FI, which is plotted at the end of the window. For example, time steps 1–8 could represent data from years 2001 to 2008. For this example, we assign the value of FI to time step 8 (e.g. 2008). The next time window will go from time 2–9 (e.g. 2002–2009), followed by time step 3–10, etc.

**Table 1.** Sample data for a time window.

time step	*Y*_1_	*Y*_2_
1	0.6	1.5
2	2	1.5
3	0.3	1
4	3.5	4.8
5	0.95	2
6	3.1	4
7	2.4	1.8
8	2.7	2.1
Δ*Y*	0.5	1

When all the points are binned together, then probability distribution (*p_i_*) for each window is estimated by using the following equation [16]:

3.3 $$p_{i}=\frac{\text{number}\;\text{of}\;\text{points}\;\text{in}\;\text{state}}{\text{total}\;\text{number}\;\text{of}\;\text{point}\;\text{in}\;\text{window}}.$$

**Figure 2.**
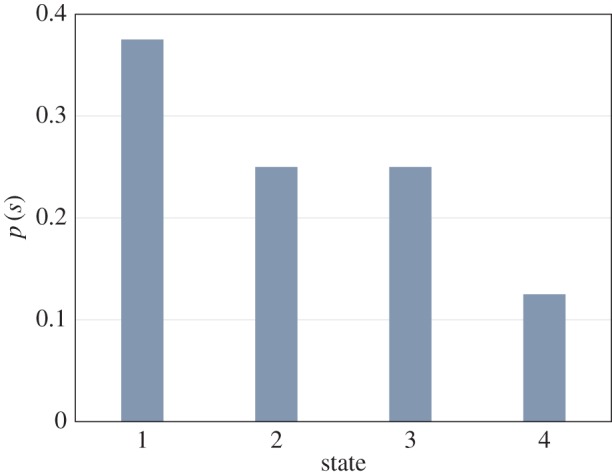
Probability distribution.

**Figure 3.**
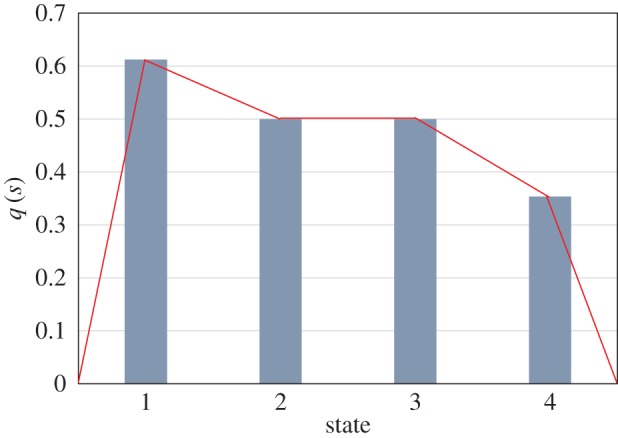
Amplitude of the probability distribution.

The probability distribution for the sample data in table 1 is shown in figure 2. Then the amplitude, *q* (*q_i_* = √*p_i_*) and FI for each window is calculated by using equation (2.4), where the initial and final *q_i_* is set as zero. Figures 2 and 3 display the *p*(*s*) and *q*(*s*) for each state based on the sample data in table 1. The FI for the sample data using equation (2.4) is 4 × [(0 − 0.61)^2^ + (0.61 − 0.5)^2^ +(0.5 − 0.5)^2^ + (0.5 − 0.35)^2^ + (0.35 − 0)^2^] = 4 × (0.375 + 0.13 + 0 + 0.21 + 0.125) = 4 × 0.534 = 2.136.

In practice, system variables fluctuate such that not all the variables meet the size of state criteria. Therefore, a new parameter called tightening level (TL) is introduced to adjust the binning criteria. The TL adjusts the binning criteria such that a point can be declared to be within a given hyper-rectangle (a particular state of the system) when a certain percentage of the variables meet the size of states criteria [10]. For example, if a system is characterized by 100 variables and 95 of the variables indicate that a particular point fits within the state being evaluated, then the two points would be binned together at a 95% TL. There are no specific criteria for setting the TL, hence we take the average of all the TLs from strict (TL = 100%) to the lowest TL in which more than one state is observed in a window [10]. Moreover, to focus on the trends in dynamic order and not on fluctuations, we may report a smoothed FI by averaging neighbouring FI values [24]. For example, if a time step of 3 is chosen for the block average, then three consecutive FI values (e.g. FI_1_, FI_2_ and FI_3_) are averaged, and that average value acts as the representative for all the three consecutive FI values. The averaging is essentially a high frequency filter. Note the number of years including the averaging has to be smaller to capture more accurate trends in dynamic order.

## 4. Interpretation of Fisher information

The sustainable regimes hypothesis was developed to provide a construct for interpreting FI [11,13,17].

— A system is considered to be in an orderly dynamic regime when a non-zero FI remains nearly constant over time (i.e. d〈FI〉/d*t* ≈ 0).
— A steady decrease in FI indicates that the system is losing its order, functionality, stability and the patterns are breaking down. This declining trend may provide warning of an impending regime shift [22] or even a catastrophe, but the index alone will not pinpoint any particular indicator that contributes to the shift. Potential drivers may be identified post hoc using approaches (e.g. Spearman's rank order correlations [24,26]).
— A steady increase in FI indicates that the system is becoming more organized/stable.
— A sharp decrease in FI indicates a regime shift and the intensity of the shift is related to the depth of the drop of FI [11].

Further, researchers have noted that the actual FI value is not as important as the ability of the system to remain within a desired regime. Accordingly, when comparing different regimes, note that a stable system regime has a relatively high and stable mean FI (*μ*FI) and low standard deviation in FI (*σ*FI) [18,21] than others.

Researchers have studied the behaviour of FI in the neighbourhood of a tipping point [29]. While most systems tend to exhibit declining FI as a warning of impending transitions [21,22], a number of theoretical scenarios have been explored to model expected behaviour under different conditions [22]. From this study, it is clear that the behaviour of FI depends heavily on the trends in the variables as the system approaches a tipping point.

## 5. Case study

To illustrate the use of FI for assessing system stability, we assessed the evolution of FI as global-mean temperature changed from 1880 to 2015. The data were collected from the National Aeronautics and Space Administration (NASA), Goddard Institute for Space Studies [31]. The data included monthly global temperature anomalies in 0.01°C from the base period of 1950 to 1980 (i.e. how average monthly temperatures diverge from average temperatures recorded from 1950 to 1980). In order to assess how the average temperatures evolved over time, we organized the time series data such that each month represents one system variable and end up with 12 variables describing global temperature anomalies from January to December for each time step (year). The rationale is that if the climate is stable, the temperature of each of the months would be about the same every year regardless of seasonal variations. For this analysis, the window size of 40 years is chosen, as changes in climate tend to transpire rapidly over only a few decades (e.g. 40 years) [16,32,33]. Using the approach described previously, the size of state is calculated and found to be 35.96, 39.21, 34.14, 31.17, 32.94, 24.68, 24.14, 31.28, 25.87, 36.43, 27.25 and 40.81, respectively, for the 12 variables (i.e. global temperature anomalies from January to December) used for the analysis. The Python scripts supplied at https://github.com/csunlab/fisher-information (accessed 10 July 2016) were used to compute FI for this study.

**Figure 4.**
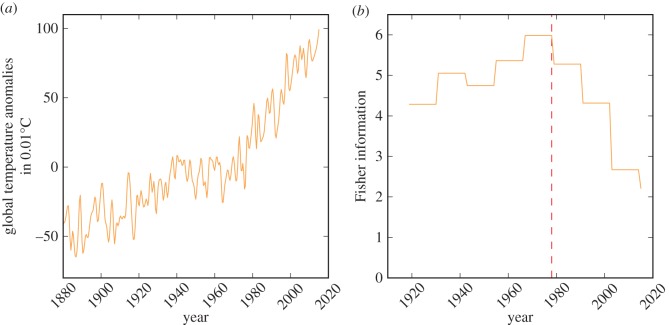
Evolution of (*a*) global-mean temperature from 1880 to 2015 and (*b*) FI for global-mean temperature.

Figure 4*a* shows the evolution of the global-mean temperature from 1880 to 2015 and figure 4*b* provides the FI for the corresponding data. A window size of 40 is chosen, a moving window increment of 1 is used and FI is assigned at the end of the window; therefore, the first value reported is for 1919, representing 1880 to 1919. From the figure, we observe a significant change in FI from 1978 with continual decrease since then, suggesting a rapid change in the global-mean temperature. Moreover, the FI for the period of 1919 to 1978 was more stable with an average (*µ*FI) of 5.09 and standard deviation (*σ*FI) of 0.89, than the period of 1979 to 2015 (*µ*FI = 4.04, *σ*FI = 1.32). The decline in FI from 1979 to 2015 represents a 62.62% change indicating significant variation in global temperature patterns.

Naturally, these analyses are not sufficient to fully capture how the global climate is performing, however, the change in the FI trajectory during the late 1970s corresponds with the period in which our global societal demand (ecological footprint) also began to surpass the global biocapacity to supply that demand [34]. Moreover, the latter part of the twentieth century is also noted for major anthropogenic global environmental impacts [35] and studies identify this period as the base of a new Anthropocene epoch [36].

## 6. Conclusion

The main objective of this work was to present FI as a useful method for data mining applications by demonstrating its use in assessing patterns in complex system data [37]. FI has been applied to a variety of systems but the transferability and use of the method have been hindered by the algorithm development in Matlab with deployed applications available only by contacting the code developer directly. The creation of an open access Python script offers significant opportunities for the general scientific community to facilitate the calculation of FI for any multivariate data. The assessment of global temperature provides a simple case study and suggests that it appears to have destabilized in the latter part of the twentieth century (i.e. since 1978) which corresponds to increasing ecological demand, declining biocapacity, and the initial stages of the new Anthropocene. This case study afforded the ability to demonstrate the power of the index and shows how FI can provide information about trends in complex system behaviour. This effort showcased FI as a viable tool for mining data. By providing public access to the Python script for FI, we hope to expand use of the method to a broader audience who may be interested in methods for detecting hidden trends and identifying signals useful for system evaluation and management.

## Data accessibility

The data were collected from the National Aeronautics and Space Administration (NASA), Goddard Institute for Space Studies for this study. The Python library to compute FI can be freely accessed from GitHub at https://github.com/csunlab/fisher-information.
